# *Ureaplasma urealyticum* disseminated multifocal abscesses in an immunocompromised adult patient: a case report

**DOI:** 10.1186/s12879-020-4771-z

**Published:** 2020-01-15

**Authors:** Carolina Diaz Pallares, Thomas Griener, Stephen Vaughan

**Affiliations:** University of Calgary Cumming School of Medicine, 3330 Hospital Dr NW, Calgary, AB T2N 4N1 Canada

**Keywords:** *Ureaplasma urealyticum*, Multifocal abscesses, Adult patient, Case report

## Abstract

**Background:**

*Ureaplasma urealyticum* is a fastidious bacteria which lacks a cell wall. Extragenital infections are rare in immunocompetent adults. There are few literature reports of perinephric abscess. We present a case of non-resolving multifocal “culture-negative” abscesses in a hypogammaglobulinemic adult female due to *U*. *urealyticum.*

**Case presentation:**

66-year-old female with a one-week history of fever, malaise and new right hip and leg pain. Past medical history was notable for chronic pancytopenia secondary to in remission B cell follicular lymphoma, ESRD on intermittent hemodialysis with bilateral nephrostomy tubes and Crohn’s. CT abdomen/pelvis revealed a small left perinephric hematoma and proximal right femur fluid collection. Persistent right thigh pain led to additional ultrasound with anterior thigh collection and CT revealed an irregular rim-enhancing fluid collection in the left posterior pararenal space. Antimicrobial therapy included ertapenem and vancomycin followed by meropenem, trimethoprim-sulfamethoxazole, daptomycin and metronidazole in setting of persistent culture-negative results and clinical deterioration. Following detection of *U. urealyticum* by 16S rDNA PCR in both left pararenal and right trochanteric bursa abscesses doxycycline was started. Despite this, the patient died four days later.

**Conclusions:**

Disseminated infection by *U*. *urealyticum* has been documented in immunocompromised adult patients with few reports of perinephric abscess. We propose that ascending genitourinary route led to perinephric abscess. The multiple disseminated fluid collections make it highly suspicious for hematogenous spread given the lack of radiographic enhancement to suggest contiguous spread. Diagnosis and treatment of *U*. *urealyticum*-disseminated infection is extremely challenging as culture is laborious and not routinely performed. Furthermore, the lack of cell wall renders beta-lactams and vancomycin ineffective and therefore requirement for “atypical” coverage. Early diagnosis and treatment are key to prevent further complications and death.

## Background

*Ureaplasma urealyticum* is a fastidious bacteria which lacks a cell wall. It is notable for having a small genome and adheres to the mucosa of the urogenital tract of adults or respiratory tracts in infants [1]. It primarily resides extracellularly, but intracellular localization has been described [2]. *Ureaplasma* species are normal genital flora of sexually experienced adults [3] with 40 to 80% of healthy women [1] having vaginal colonization [4]. It causes a variety of diseases in neonatal populations and adult urogenital disease [5, 6]. Extragenital infections are rare in immunocompetent adults [7]. In immunodeficient patients it may cause a more severe disease. Current literature demonstrates only a few reports of *U. urealyticum* as the cause of disseminated disease in this population [8]. We present a case of non-resolving multifocal “culture-negative” abscesses in a hypogammaglobulinemic adult female due to *U*. *urealyticum.*

## Case presentation

66-year-old female with a one-week history of fever, malaise and confusion admitted with a diagnosis of sepsis NYD.

Medical history was notable for chronic pancytopenia secondary to CD10 positive B cell follicular lymphoma diagnosed in 2013 treated with induction bendamustine and maintenance Rituximab until December 2015 with a bone marrow biopsy in 2017 showing hypocellularity without disease activity, ESRD secondary to obstructive uropathy from retroperitoneal lymphoma on intermittent hemodialysis with bilateral nephrostomy tubes and Crohn’s disease diagnosed in 2004 stable on Humira which was discontinued when diagnosed with lymphoma (2013). Furthermore, she had persistent hypogammaglobulinemia (IgG 5.8 g/L, IgM 0.27 g/L) and recurrent *Clostridium difficile* infections requiring fecal microbiota transplant in 2017, and in 2019 a recent admission to hospital with *Citrobacter* *freundii* and *Stenotrophomonas maltophilia* cultured from nephrostomy tube, as well as *Bacteroides fragilis* bacteremia of unidentified source treated as an outpatient with a 28-day course of IV ertapenem and oral levofloxacin.

She returned to hospital 19 days after completion of her antibiotic course with new right hip and leg pain. Aside from being febrile, vital signs were stable. Laboratory results revealed a C-reactive protein of 275 mg/L and neutrophil nadir 0.4 × 10 [9]/L. CT abdomen/pelvis revealed a small left perinephric hematoma (2.9 × 1.2 × 5.8 cm) and hypodense area surrounding the proximal right femur suggestive of fluid collection (5.1 × 2.9 cm). Ultrasound guided drainage showed a separate right greater trochanteric bursa abscess (6.3 × 6.7 × 4.8 cm). Both aspirates consistent with purulent material when drained via catheter. Repeat ultrasound for persistent right thigh pain, performed 5 days later showed an additional collection along the anterior thigh proximally within the deep quadriceps’ muscles (8.3 × 3.4 × 22.6 cm). Purulent fluid was drained, and a catheter placed. Gram stain of these abscess fluids revealed heavy neutrophils but no organisms. Anaerobic and aerobic bacterial culture, fungal and mycobacterial culture were negative, as were multiple blood and urine cultures.

Clinical deterioration followed with decreased level of consciousness requiring intubation and ICU admission. Repeat CT imaging 15 days post-admission revealed an irregular rim-enhancing fluid collection in the left posterior pararenal space (2.5 × 2.9 × 5.3 cm) with moderate inflammatory stranding in the left perinephric and pararenal space requiring aspiration and drain insertion. Also, a new fluid collection (2.2 × 4.6 cm) in the medial aspect of the right gluteus maximus muscle was identified. There was minimal decrease in size of the right greater trochanter bursa abscess despite drainage. On day 22, left pararenal and right trochanteric bursa abscess specimens were positive for *U*. *urealyticum* by a laboratory-developed broad-range 16S rDNA PCR using dual-priming oligonucleotide (DPO) primers [9]. DNA was extracted from patient specimens using the QIAamp DNA Mini Kit. Identification was performed by Sanger sequencing of the 16S product using the same DPO primers and the resulting sequence was queried against the IDNS bacterial database (Smartgene IDNS, Switzerland). The resulting 16S amplicon was 468 basepairs in length and matched with 99.79% identity to *U. urealyticum* ATCC 33699.

Initial antimicrobial therapy included ertapenem and vancomycin. In setting of persistent culture- negative results and clinical deterioration, therapy was modified to meropenem, trimethoprim-sulfamethoxazole, daptomycin and metronidazole was added given her history of *B. fragilis* bacteremia. Following detection of *U. urealyticum* by 16S PCR on admission day 22, doxycycline 100 mg IV twice a day was started. Despite this, the patient died four days later.

## Discussion and conclusion

Disseminated infection by *U*. *urealyticum* has been documented in adult patients with hypogammaglobulinemia, usually associated with arthritis [10, 11], rarely osteomyelitis [12] and one case of brain abscess [7]. There are few literature reports of perinephric abscess; in a transplanted kidney and pyelonephritis [13].

**Fig. 1:**
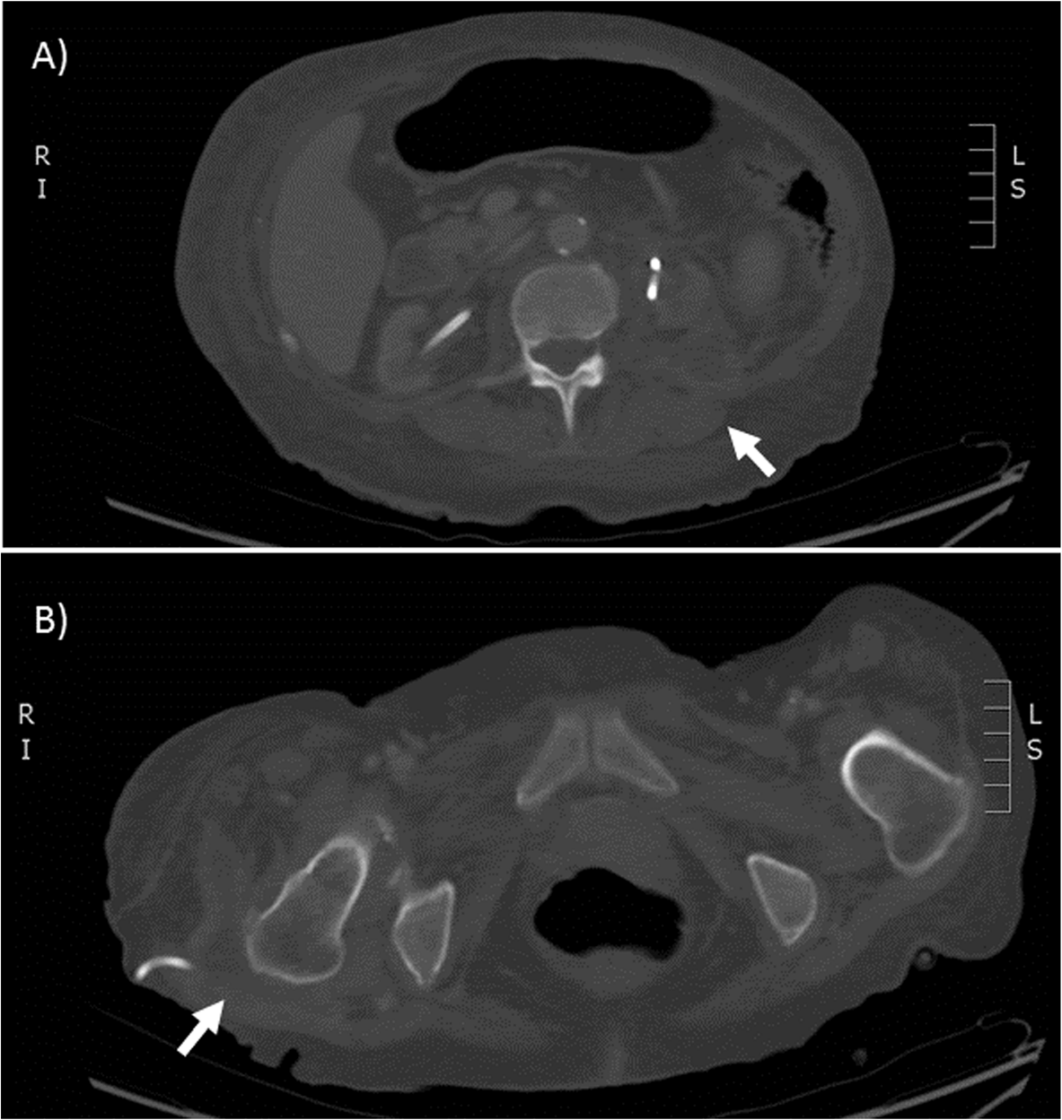
**a**) Irregular rim-enhancing fluid collection in the left posterior pararenal space. **b**) Large complex fluid collection in the right greater trochanter bursa

We propose that ascending urinary route led to perinephric abscess followed by the multiple surgical manipulation of the patient’s bilateral nephrostomy tubes leading to hematogenous seeding. Interestingly, the multiple disseminated fluid collections: left perinephric and pararenal space (Fig. 1-a), right greater trochanter bursa (Fig. 1-b) and the anterior right thigh quadriceps muscle, make it highly suspicious for hematogenous spread given the lack of radiographic enhancement to suggest contiguous spread from the perinephric space to trochanteric bursa.

Diagnosis and treatment of *U*. *urealyticum* disseminated infection is extremely challenging. Culture of *Ureaplasma* is laborious and not routinely performed in most clinical microbiology laboratories as specialized media is preferred. Targeted molecular techniques (e.g. PCR) have been developed, but are also not widely available [14, 15]. Where available, culture is more cost effective than molecular testing and provides an isolate to perform antimicrobial susceptibility testing [11, 5].

It is important to note that the lack of cell wall renders beta-lactams and vancomycin ineffective and therefore requirement for “atypical” coverage is necessary. Several regimens have been suggested including doxycycline, macrolide and occasionally quinolones. We initiated treatment with doxycycline as the patient had a recent prolonged course of levofloxacin. Antibiotic susceptibility molecular resistance testing was not available; however, doxycycline is generally considered to be active against *U*. *urealyticum* with reported low MIC values compared to other multiple agents [16].

Patients with antibody deficiency demonstrate unique susceptibility to infection with *U. urealyticum*. We describe a hypogammaglobulinemic patient with fatal *U. urealyticum*-disseminated infection recovered from multiple abscesses with initial negative cultures and failing standard therapy. Awareness of such unusual infections in this population is key to an early diagnosis and treatment to prevent further complications and death.

## Data Availability

All data and materials of this article are included in the manuscript.

## Abbreviations

ESRD: End stage renal disease
ICU: Intensive care unit
NYD: Not yet diagnosed
PCR: Polymerase chain reaction
*U*. *urealyticum*: *Ureaplasma urealyticum*
